# Revisiting time to blood culture positivity: can we decrease antibiotic exposure in the NICU?

**DOI:** 10.1038/s41372-026-02629-6

**Published:** 2026-04-07

**Authors:** Rachel J. Graf, Amy Edwards, Moira A. Crowley, Devashis Mukherjee, Rita M. Ryan

**Affiliations:** 1 Department of Pediatrics, Rainbow Babies & Children’s Hospital, Case Western Reserve University, University Hospitals, Cleveland, OH USA; 2 Division of Pediatric Infectious Disease, Rainbow Babies & Children’s Hospital, Case Western Reserve University, University Hospitals, Cleveland, OH USA; 3 Division of Neonatology, Rainbow Babies & Children’s Hospital, Case Western Reserve University, University Hospitals, Cleveland, OH USA

**Keywords:** Bacterial infection, Outcomes research

## Abstract

**Objective:**

We aimed to investigate the time to positivity of blood cultures in our NICU to determine if we can better inform empiric antibiotic duration decisions in both early-onset (≤72 h) and late-onset (>72 h) sepsis evaluations.

**Study design:**

Retrospective review of all positive blood cultures isolated from infants admitted to the Rainbow Babies and Children’s Hospital NICU from July 1, 2018 to July 1, 2023.

**Result:**

There were 264 positive blood cultures from 125 infants with a median postmenstrual age of 30.9 weeks. The vast majority (86%) of positive cultures were obtained during the late-onset sepsis period. Gram-negative bacteria grew faster than gram-positive bacteria. Overall, 84% of blood cultures for all pathogenic bacteria (excluding coagulase-negative staph, fungi, and likely contaminants) were positive by 24 h.

**Conclusion:**

Our data do not support discontinuing empiric antibiotics at 24 h as our a priori target of 90% positivity was not met. These results underline the importance of evaluating an individual NICU’s data before implementing a practice change.

## Background

Sepsis is a significant cause of morbidity and mortality in neonates. Thus, empiric antibiotics are routinely started pending blood culture results. However, prolonged antibiotic durations are associated with neonatal morbidity. Specifically, increased antibiotic exposure (even 4–7 days vs. 1–3 days) has been associated with increased neurodevelopmental impairment, bronchopulmonary dysplasia, retinopathy of prematurity, and necrotizing enterocolitis in preterm infants [1–9]. In term infants, increased antibiotic exposure has been associated with delayed initiation of breastfeeding, obesity, recurrent wheezing, gastrointestinal disorders, and autism [10–14]. These associations have prompted antimicrobial stewardship programs at neonatal centers to investigate the risk-benefit ratio of decreasing the length of empiric antibiotic therapy from the routine 48 hours (h) of negative bacterial cultures to a shorter 24–36 h duration [15–20]. However, these studies have been conducted at single centers and do not accurately reflect the diverse neonatal population.

When comparing infants undergoing early-onset sepsis evaluation, Sanchez and colleagues found that those exposed to antibiotics for only 24 h were not more likely to have antibiotics restarted within the first week of life [15]. Those who were exposed to 48 h of antibiotics were more likely to have antibiotics reinitiated within 7 days of discontinuation [15]. Overall mortality before discharge did not differ between the two groups in this study [15]. However, when Fleiss et al. analyzed these data from Sanchez et al., they noted in their commentary that sepsis-related mortality was higher in the 24 h group with an odds ratio of 7.90 with a wide 95% CI = (0.41, 154.0) [21].

Based on previous antibiotic stewardship quality improvement work, our neonatal intensive care unit (NICU) currently discontinues empiric antibiotics after 36 h of negative bacterial cultures for both early-onset sepsis (EOS, defined as onset ≤72 h of life) and late-onset sepsis (LOS, defined as onset >72 h of life) evaluations. Given recent evidence of morbidities associated with prolonged antibiotic exposure in neonates, we investigated whether we could shorten the duration of empiric antibiotic exposure by examining time to positivity (TTP) from collection for all positive bacterial blood cultures. We investigated the association between clinical, microbiological, and demographic factors and earlier versus later TTP. The overall goal was to inform our clinical decision for empiric antibiotic duration for both EOS and LOS evaluations with the hope of decreasing antibiotic exposure time and doses.

## Methods

This study was approved by the University Hospitals’ Institutional Review Board with waiver of informed consent. All methods were performed in accordance with the relevant guidelines and regulations. All positive blood cultures isolated from infants admitted to the Rainbow Babies and Children’s Hospital NICU from July 1, 2018 to July 1, 2023 were included. This list of neonates was identified by querying our hospital’s database of positive blood cultures. Pertinent demographic, clinical, laboratory, and outcome data were obtained from infants’ electronic health records and stored in a secure REDCap database.

Our EOS protocol advises obtaining blood (at least 1 mL for any blood culture) for culture from either a peripheral sample or at the time of insertion of umbilical lines. Our EOS protocol utilizes ampicillin and gentamicin. Ceftazidime is added if a baby was born to a mother with preterm premature rupture of membranes before 34 weeks GA and greater than 18 h and/or has a history of an ampicillin-resistant infection during pregnancy. Our LOS protocol suggests obtaining two peripheral blood cultures, or one peripheral and one from a central line if applicable. Neonatal peripherally inserted central catheters less than 2 Fr in size are not cultured, but other central lines may be. Our LOS protocol utilizes nafcillin and gentamicin. Vancomycin is given instead of nafcillin if there is a central line, a history of methicillin-resistant Staphylococcus aureus, or symptoms of shock. Peripheral blood cultures are obtained via arterial puncture by physicians and advanced practice providers, and any culture from a central line (except initial umbilical line cultures) is drawn by a nurse on our central line team. Only aerobic cultures are sent. We did not look at cerebrospinal fluid cultures as the EOS and LOS protocols only mandate lumbar puncture after a positive blood culture result, which would be after the patient was started on antibiotics.

Stata 15.0 (StataCorp, College Station, TX) was used for statistical analysis. The Wilcoxon rank sum test was used to compare non-normally distributed continuous variables between two groups. Fisher’s exact test was used for categorical variables. The Kruskal–Wallis test with post hoc Dunn’s test was used for nonparametric continuous variable analysis with more than two groups. Linear regression was used to compare two continuous variables. Kaplan-Meier “survival” curves were utilized to compare TTP between groups. Log rank test was used to compare survival curves. Confidence intervals for binary proportions were done using the Clopper–Pearson interval. We did not correct for general multiple comparisons.

Although we initially analyzed all culture results, we also analyzed a subset of cultures positive for organisms routinely covered by standard “first-line” antibiotic choices. Thus, the following organisms were later removed from our analysis as they are either not bacterial pathogens, are often considered contaminants, or are not covered for in our typical sepsis protocols: *Candida albicans, Candida parapsilosis*, Coagulase-negative Staphylococcus (CoNS*), Coryneform bacterium, Cyberlindnera*, Gram-positive *bacilli*, and *Micrococcus*. We acknowledge that CoNS can certainly be a pathogen, but it is often not covered in some NICUs when a typical sepsis evaluation is initiated. In addition, CoNS tends to be a less aggressive organism. Thus, vancomycin coverage is often not initiated until a blood culture returns positive for gram-positive (GP) cocci.

## Results

Over the five-year study period, 264 positive blood cultures were isolated from 125 infants with 79 infants (63%) having only one positive blood culture. The remainder had 2 to 11 positive blood cultures, many of which were serially positive within the same sepsis episode. Among infants with positive blood cultures, 64% were male, and 56% were Black (Table 1). Males were significantly overrepresented in the cohort of infants with any positive culture when compared with 50% male standard (*P* < 0.05 by Fisher’s exact test; Table 1). The cohort of infants with any positive culture had a median gestational age (GA) of 26.6 weeks and a median birth weight of 920 grams. The median postmenstrual age (PMA) at the time a positive culture was drawn was 30.9 weeks (IQR: 27.4, 39.4), and the median age was 20 days (10, 55). The median age at the time of the first positive culture was 14 days. The majority (86%) of positive cultures were collected after 72 h of life, representing LOS. Only 14% of positive cultures were in the EOS time frame. Of the first positive cultures, only 24% were collected before 24 h of age. Their median TTP was 21 h (16, 31) (Table 2). Higher gestational age was associated with decreased TTP (*P* < 0.02 for any culture, <0.04 for first culture, by linear regression; Table 1). We found that each 0.4 week decrease in gestational age was associated with one hour longer TTP.

**Table 1:** Clinical and demographic characteristics of 125 NICU infants with 264 positive blood cultures.

No. of infants/No. of positive blood cultures	125/264
Male^a^	80 (64%)
Birth weight (grams)	920 (650, 1650)
Gestational age^b^ (weeks)	26.6 (24.5, 31.3)
Postmenstrual age (weeks) at time culture drawn	30.9 (27.4, 39.4)
Age at first positive culture (days) (*n* = 125)	14 (2, 33)
Age at any positive culture (days) (*n* = 264)	20 (10, 55)
Race:	
White	37 (30%)
Black	70 (56%)
Mode of delivery:	
Cesarean	64 (54%)
Vaginal	55 (46%)
Duration of maternal rupture of membranes (hours)	0 (0, 29)
Maternal GBS positive	15 (12%)
Maternal intrapartum antibiotics	85 (69%)
Maternal intrapartum fever^c^	16 (13%)
Repeat positive culture after initial positive culture	46 (37%)
Central line at time of first positive culture	
UVC	29 (23%)
PICC	39 (31%)
No central line	57 (46%)

Data presented as median (IQR) or *N* (%).

*TTP* time to positivity, *UVC* umbilical venous catheter, *PICC* peripherally inserted central catheter.

^a^Males were significantly overrepresented in the cohort of infants with any positive cultures (compared with 50% male standard, *P* < 0.05 by Fisher’s exact test).

^b^Higher GA was associated with decreased TTP (*P* < 0.02 for any culture, <0.04 for first culture, by linear regression).

^c^Maternal intrapartum fever was associated with decreased TTP (*P* < 0.002 for any culture, <0.004 for cultures < 7 days, <0.002 for cultures < 72 h by Wilcoxon rank sum test).

**Table 2:** Microbiological characteristics of positive NICU blood cultures.

Type of culture/microorganism	*N* (%)	Time to positivity (hours)^a,b^	Positive by 24 h (%)
No. of positive blood cultures^a^	264	21 (16, 31)	60%
All significant pathogens^c^	146	17 (13, 22)	84%
Gram-positive organisms	135	22 (18, 32)	56%
Selected Gram-positive organisms^c^	54	18 (15, 22)	80%
Gram-negative organisms	92	16 (13, 19)	86%
CoNS, fungus, contaminants	118	31 (23, 38)	68%
**Early** **onset** **sepsis (≤72 h):**	36 (14%)	17 (13, 27)	69%
Gram-positive organisms	16	23 (13, 29)	44%
Selected Gram-positive organisms^c^	5	14 (12, 18)	60%
Gram-negative organisms	20	14 (11, 17)	90%
CONS, fungus, contaminants	11	31 (23, 68)	36%
**Late onset sepsis (>72 h):**	228 (86%)	22 (17, 32)	59%
Gram-positive organisms	119	22 (18, 32)	58%
Selected Gram-positive organisms^c^	49	19 (15, 22)	83%
Gram-negative organisms	72	17 (13, 20)	85%
CoNS, fungus, contaminants	107	31 (23, 38)	31%
Microorganism^b^			Mortality (*n*/*N*)
*Candida*	31 (11%)	34 (29, 37)	0/4 (0%)
Coagulase-negative Staphylococcus (CoNS)	74 (28%)	27 (20, 36)	3/41 (7%)
*Escherichia coli*	37 (14%)	14 (12, 17)	11/33 (33%)
Enteric organisms (excluding *E. coli*)	47 (18%)	17 (13, 20)	0/16 (0%)
*Streptococcus agalactiae*	4 (2%)	12 (10, 16)	1/3 (33%)
Non-enteric Gram-negative bacilli	9 (3%)	18 (17, 24)	1/5 (20%)
*Staphylococcus aureus*	45 (17%)	19 (15, 22)	2/11 (18%)
Other	17 (6%)	34 (31, 50)	1/12 (8%)

Data presented as median (IQR) or *N* (%).

^a^Significant difference in TTP between early onset sepsis and late onset sepsis (*P* < 0.05 by Wilcoxon rank sum test)

^b^Significant difference in TTP among the different microorganism categories (*P* < 0.0001 by Kruskal–Wallis test); post-hoc Dunn test confirmed multiple differences in TTP.

^c^CoNS, fungi, and contaminants removed including Candida albicans, Candida parapsilosis, Coagulase-negative Staphylococcus (CoNS), Coryneform bacterium, Cyberlindnera, Gram-positive bacilli, and Micrococcus.

*TTP* time to positivity.

### Maternal effects on time to positivity (TTP)

**Fig. 1:**
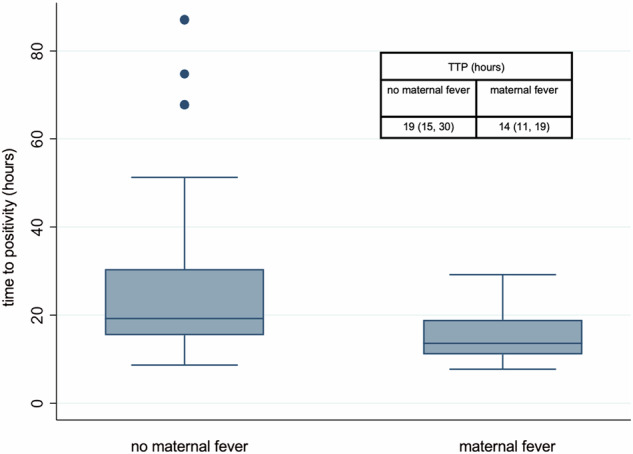
Time to positivity for positive cultures in the first week of age, born to babies without vs with maternal fever. *P* < 0.004 by Wilcoxon rank sum test.

In this cohort of infants with positive blood cultures, 54% were born via cesarean delivery, and 46% born via vaginal delivery. The median length of maternal rupture of membranes was 0 h (0, 29). The mode of delivery did not influence TTP. Among infants with a positive blood culture, 13% of their mothers had intrapartum fever. Maternal intrapartum fever was associated with decreased TTP for any positive culture, for EOS cultures, and for cultures drawn in the first seven days of life (*P* < 0.004 for all by Wilcoxon rank sum test; Table 1; Fig. 1). We reasoned maternal fever would most likely affect TTP for cultures drawn in the first seven days after birth, so we examined that cohort. For cultures positive in the first week of life, infants with maternal intrapartum fever had a median TTP of 14 h (11, 19) compared to a median of 19 h (15, 30) for infants with no maternal intrapartum fever. Infants born to mothers with intrapartum fever, 90% had cultures grow positive within 24 h. Higher gestational age was associated with maternal fever (*P* < 0.05 by the Wilcoxon rank-sum test). When evaluating TTP in the subgroup of term infants with maternal fever who develop EOS, maternal fever was associated with shorter TTP (*P* < 0.002, by Wilcoxon rank-sum). However, gestational age was not. Of the 125 mothers, 85 (69%) received antenatal antibiotics.

### Neonatal associations with time to positivity (TTP)

At the time of a positive blood culture, 23% of the infants had an umbilical venous catheter in place, one-third had a peripherally inserted central catheter, and 46% had no central line. The presence of an umbilical venous catheter was not associated with increased TTP in babies with positive cultures in the first 7 days of life (*P* = 0.79 by Wilcoxon rank-sum test). There was no difference in time to positivity for blood cultures obtained from infants with an umbilical venous catheter, a peripherally inserted catheter, or no central line (data not shown). None of the classic markers of infection (elevated C-reactive protein, leukocytosis, increased immature neutrophils to total neutrophils, or low absolute neutrophil count at birth), at 24 h after birth, or within 12 h of a positive culture, were associated with increased TTP (data not shown).

### TTP breakdown by GP vs GN and EOS vs LOS

**Fig. 2:**
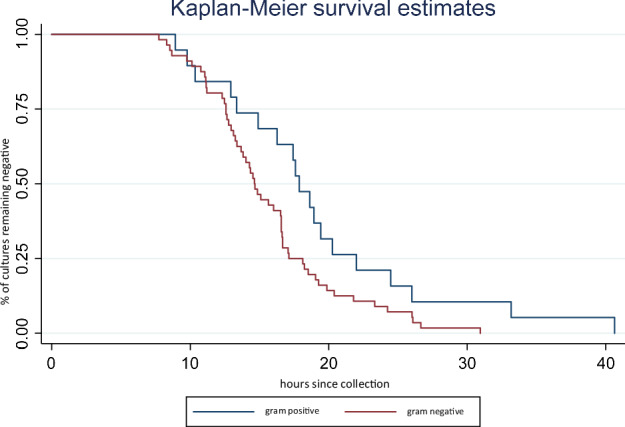
Time to positivity for Gram-negative vs. Gram-positive Organisms without CoNS and likely contaminants. *P* < 0.04 by log-rank test.

The median TTP for all positive cultures was 21 h (Table 2). Only 60% of cultures were positive by 24 h, with 84% positive by 36 h and 100% positive by 48 h (Table 3). When CoNS, fungal organisms, and contaminants were removed from the data set, the proportion of blood cultures in this “all significant pathogens” group that were positive by 24 h increased from 60% to 84% across all cultures. There was also a decrease in median TTP for this group to 17 h. TTP significantly differed between gram-negative (GN) and GP organisms (*P* < 0.04 by log-rank test; Fig. 2) when evaluating only the “significant pathogens.” There was also a significant difference in TTP between early onset sepsis and late onset sepsis (*P* < 0.05 by Wilcoxon rank sum test; Table 2).

**Table 3:** Proportion of positive NICU blood cultures at 24 h and 36 h.

Population	% pos at 24 h	95% CI	% pos at 36 h	95% CI
All positive cultures (cxs) (*N* = 264)				
All positive cxs (*n* = 264)	60%	0.54–0.66	84%	0.80–0.89
All GP cxs (*n* = 135)	56%	0.47–0.64	80%	0.720.86
All GP cxs excluding CoNS, fungi, and other likely contaminants (*n* = 54)	80%	0.66–0.89	98%	0.90–0.99
All GN cxs (*n* = 92)	86%	0.77–0.92	96%	0.89–0.99
All EOS cxs (collected <72 h age) (*n* = 36)	69%	0.52–0.84	86%	0.71–0.95
All LOS cxs (collected >72 h age) (*n* = 228)	59%	0.52–0.65	84%	0.79–0.89
*Candida* cxs (*n* = 31)	16%	0.05–0.34	71%	0.52–0.86
Coagulase-negative Staphylococcus cxs (CoNS) (*n* = 74)	43%	0.32–0.55	73%	0.61–0.83
*Escherichia coli* cxs (*n* = 37)	95%	0.82–0.99	100%	–
Enterics excluding *E. coli* cxs (*n* = 47)	81%	0.67–0.91	91%	0.80–0.98
Non-enteric GN cxs (*n* = 9)	78%	0.40–0.97	100%	–
Staph aureus cxs (*n* = 45)	82%	0.68–0.92	100%	–
*Streptococcus agalactiae* cxs (*n* = 4)	100%	–	100%	–
Other cxs (*n* = 17)	6%	0.00–0.29	53%	0.28–0.77
Only each infant’s first positive culture (*n* = 125)				
All 1st positive cxs (*n* = 125)	66%	0.57–0.74	82%	0.75–0.89
All GP cxs (*n* = 66)	45%	0.33–0.58	68%	0.56–0.79
All GP cxs excluding CONS, fungi, and other likely contaminants (*n* = 18)	72%	0.47–0.90	94%	0.73–0.99
All GN cxs (*n* = 54)	93%	0.82–0.98	100%	–
All EOS cxs (collected <72 h age) (*n* = 35)	69%	0.51–0.83	86%	0.70–0.95
All LOS cxs (collected >72 h age) (*n* = 90)	64%	0.54–0.74	81%	0.71–0.89
CoNS cxs (*n* = 41)	41%	0.26–0.58	66%	0.49–0.80

*CI* confidence interval, *GP* Gram-positive, *CoNS* coagulase-negative staphylococcus, *GM* Gram-negative, *EOS* early-onset sepsis, *LOS* late-onset sepsis, *h* hours.

^a^All row categories had 100% positive by 48 h.

Overall, for all GP bacteria excluding CoNS and contaminants, the TTP was 18 h (15, 22) with 80% positive by 24 h. When broken down by sepsis time frame, 60% of GP organisms were positive by 24 h in EOS, with a TTP of 14 h (12, 18). Eighty-three percent of GP organisms were positive by 24 h in LOS with a TTP of 19 h (15, 22). Similarly, for all GN bacteria excluding contaminants, 86% were positive by 24 h with a TTP of 16 h (13, 19). Ninety percent of GN organisms were positive by 24 h in EOS with a TTP of 14 h (11, 17) and 85% positive by 24 h in LOS with a TTP of 17 h (13, 20).

**Fig. 3:**
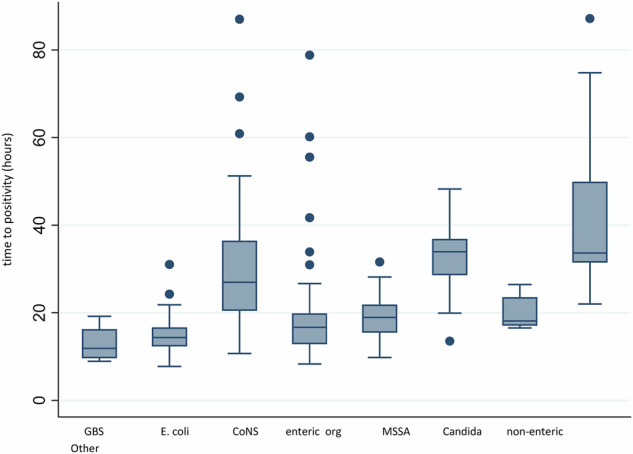
Time to positivity of different microorganisms. *P* < 0.0001 by Kruskal–Wallis test.

There was a significant difference in TTP among the different microorganism categories (*P* < 0.0001 by Kruskal–Wallis test; Fig. 3). Post-hoc Dunn test identified these multiple differences in TTP. CoNS had the longest TTP of 27 h (20, 36), and *Streptococcus agalactiae* (GBS) had the shortest TTP of 11 h (10, 16). Candida species had a TTP of 34 h (29, 37). Sepsis-associated mortality was highest with *Escherichia coli* and GBS: 33% (11/33) and 33% (1/3), respectively.

## Discussion

Given our a priori target of 90% positivity within 24 h of culture attainment for all pathogenic bacteria, our data do not support discontinuing empiric antibiotics for neonatal sepsis at 24 h, as only 84% of blood cultures were positive for “significant pathogens” at that time. While we note that more blood cultures are generally drawn soon after birth during the EOS time period, the burden of positive NICU blood cultures is much higher in infants over two weeks of age during the LOS time period. More work is needed to develop further strategies to reduce LOS in the NICU.

In the EOS time period, 90% of GN bacteria grew positive by 24 h. Thus, GN coverage could be eliminated at 24 h in EOS. This was suggested by Durrani and colleagues, who recommended changing antibiotic coverage to target only GP organisms after 24 h in LOS [22]. This could eliminate a single dose of gentamicin for EOS if every 24 h dosing is being utilized (the second dose at 24 h of culture growth would be eliminated). However, the Rainbow Babies and Children’s NICU only administers gentamicin every 24 h for infants older than 44 weeks PMA. This could be beneficial in this limited scenario, which would have affected 13% of our LOS-positive cultures.

For LOS, Mukhopadhyay et al. found that empiric antibiotics not targeting CoNS could be safely discontinued at 36 h [16]. In terms of reducing GN coverage, they found that only 76% of GN bacteria were detected by 24 h, and were only able to reduce their GN to 36 h [16]. In our cohort, only 85% of GN bacteria were positive by 24 h in LOS, making a 24 h cut-off unattractive.

Our data showed that positive blood cultures from infants with lower GA at birth had longer TTP, although PMA and TTP were not significantly associated. We do not have information on the volume of blood obtained for each blood culture. Infants with lower GA may have smaller volumes of blood sent for EOS culture due to their smaller weight, which could account for a lower TTP. However, there was no difference in TTP by PMA. While all blood cultures obtained at the Rainbow Babies and Children’s NICU are ideally at least 1 milliliter, we did not collect data on culture volume. We acknowledge that this is a limitation of our study. To further study differences in blood culture volumes, the blood culture vials would need to be weighed individually before analysis. In Rued et al., the authors found that the volume of blood obtained did not vary among patients with culture-negative sepsis, bacteremia, or sepsis rule-out, nor did it vary by sex, chronological age, weight at the time of culture collection, or the source of the sample [23]. In their study, blood cultures obtained during the night shift were associated with smaller volumes [23]. This suggests that there may be other reasons for the effect of GA on TTP rather than the amount of blood culture volume. However, one multicenter study found no difference in TTP based on GA [20]. This study additionally found no difference in TTP between the most common organism in preterm infants, *E. coli*, and the most common organism in term infants, GBS [20]. Enhancements to our electronic medical record now include a field in the blood culture collection order to record the volume of blood obtained at the time the culture is drawn. Thus, in the future, we could evaluate whether the blood volume of the culture affects TTP in our center. At this time, we do not have a good explanation for why infants with lower GA (even when the culture is done long after birth) have a longer TTP.

Overall, our results emphasize the importance of looking at an individual NICU’s data before implementing a practice change. In some circumstances, shortening empiric antibiotic courses while awaiting blood culture results could reduce antibiotic exposure in the NICU. However, reducing the duration from 36 h to 24 h still prevents exposure to only a couple of doses of antibiotics. Conversely, treatment for “culture-negative sepsis” leads to many more neonates being exposed to many more doses of antibiotics, causing greater harm for an unclear diagnosis. Stocker states, “Fear of missing evolving neonatal sepsis is the key driver for antibiotic overtreatment early in life.” [24]. One study by Giannoni et al. found disproportionate antibiotic exposure in the first week of life compared to the number of EOS cases. For each case of EOS, 58 neonates were started on antibiotics, and 273 antibiotic days were administered [25]. A greater impact in decreasing antibiotic exposure in the NICU would be achieved if extended antibiotic exposure to treat “culture-negative sepsis” could be avoided. This may be a more worthy target for clinicians to decrease possibly unnecessary antibiotic exposure.

Our study’s limitations include the absence of blood volume data, the inclusion of serial cultures, and potential delays in the start of incubation. We did not examine how many of the mothers who received antenatal antibiotics had infants with EOS, nor how that may have affected their TTP. We acknowledge that this is a limitation of our study. In addition, this is a single-center study.

In summary, we found that gram-negative coverage could be safely discontinued at 24 h in EOS. Overall, since our target was 90%, the percentage of cultures positive by 24 h was not sufficiently high to recommend routine discontinuation of empiric antibiotics after only 24 h of culture incubation.

## Data Availability

The de-identified dataset generated from the study is available from the corresponding author on reasonable request and following approval by the Institutional Review Board of Rainbow Babies and Children’s Hospital. The study was presented in part as a poster presentation at the Pediatric Academic Societies Meeting in Honolulu, Hawaii on April 25^th^, 2025 as well as at a scientific session also at the Pediatric Academic Societies meeting in Honolulu.
